# Neutralisation of Interleukin-13 in Mice Prevents Airway Pathology Caused by Chronic Exposure to House Dust Mite

**DOI:** 10.1371/journal.pone.0013136

**Published:** 2010-10-01

**Authors:** Kate L. Tomlinson, Gareth C. G. Davies, Daniel J. Sutton, Roger T. Palframan

**Affiliations:** Immunology Research, UCB, Slough, United Kingdom; Dana-Farber Cancer Institute, United States of America

## Abstract

**Background:**

Repeated exposure to inhaled allergen can cause airway inflammation, remodeling and dysfunction that manifests as the symptoms of allergic asthma. We have investigated the role of the cytokine interleukin-13 (IL-13) in the generation and persistence of airway cellular inflammation, bronchial remodeling and deterioration in airway function in a model of allergic asthma caused by chronic exposure to the aeroallergen House Dust Mite (HDM).

**Methodology/Principal Findings:**

Mice were exposed to HDM via the intranasal route for 4 consecutive days per week for up to 8 consecutive weeks. Mice were treated either prophylactically or therapeutically with a potent neutralising anti-IL-13 monoclonal antibody (mAb) administered subcutaneously (s.c.). Airway cellular inflammation was assessed by flow cytometry, peribronchial collagen deposition by histocytochemistry and airway hyperreactivity (AHR) by invasive measurement of lung resistance (R_L_) and dynamic compliance (C_dyn_). Both prophylactic and therapeutic treatment with an anti-IL-13 mAb significantly inhibited (P<0.05) the generation and maintenance of chronic HDM-induced airway cellular inflammation, peribronchial collagen deposition, epithelial goblet cell upregulation. AHR to inhaled methacholine was reversed by prophylactic but not therapeutic treatment with anti-IL-13 mAb. Both prophylactic and therapeutic treatment with anti-IL-13 mAb significantly reversed (P<0.05) the increase in baseline R_L_ and the decrease in baseline C_dyn_ caused by chronic exposure to inhaled HDM.

**Conclusions/Significance:**

These data demonstrate that in a model of allergic lung disease driven by chronic exposure to a clinically relevant aeroallergen, IL-13 plays a significant role in the generation and persistence of airway inflammation, remodeling and dysfunction.

## Introduction

Chronic exposure of the human airway to inhaled allergen drives inflammatory processes and remodels airway structure, resulting in the development of clinical symptoms of asthma. A mouse disease model driven by chronic exposure of the airway to whole house dust mite extract (HDM) has been developed to more accurately understand the dynamic pathophysiological mechanisms linking allergen-induced airway inflammation, structural changes and dysfunction. HDM is a clinically relevant aeroallergen known to activate the immune system through pattern recognition receptors [1]–[3] and proteolytic activity [4]. The HDM model shares many pathological features with persistent human asthma, notably eosinophilic airway inflammation, mucus hypersecretion, fibrosis of the airway wall and airway hyperreactivity (AHR) to methacholine [5]. The objective of the work detailed herein was to determine the role of interleukin-13 (IL-13) in the generation and persistence of airway pathology caused by chronic exposure to HDM.

IL-13 is a Th2 cytokine structurally and functionally related to IL-4 [6]. In contrast to IL-4, IL-13 is unable to polarise T cells or induce their proliferation [7]. The central role of IL-13 is considered to be perpetuation of inflammation through the upregulation of adhesion molecules, metalloproteinases, chemokines and cytokines [8]. In asthmatic patients IL-13 mRNA and protein is elevated in bronchoalveolar lavage fluid (BAL) following airway allergen challenge [9]–[13]. Bronchial expression of IL-13 mRNA positively correlates with the number of eosinophils in BAL [12] and bronchial biopsy [11]. Furthermore, IL-13 protein is elevated in sputum from asthmatic patients, and the number of sub-mucosal cells expressing IL-13 is increased [14], [15]. Sub-mucosal cells expressing IL-13 have been identified as eosinophils [14] and mast cells [16] although many other cell types are capable of producing IL-13 in the inflamed airway. Notably, T-lymphocytes (predominantly, but not exclusively Th2 cells) represent a major source of IL-13, in addition to macrophages, basophils, dendritic cells, airway smooth muscle [7], [8], and invariant NKT cells [17].

Administration of recombinant IL-13 directly to the mouse airway, or transgenic over-expression of IL-13 in the airway, is sufficient to induce a phenotype resembling human asthma pathology, characterised by eosinophilia, airway remodelling, goblet cell upregulation, mucus hypersecretion and AHR [18]–[21]. The pharmacology of neutralising anti-IL-13 monoclonal antibodies (mAb) has been investigated in experimental models of asthma in mice [18], [22], sheep [23] and non-human primates [24], [25]. Direct evidence for a role of IL-13 in human asthma has been reported by Gauvreau *et. al.* (European Respiratory Society, Berlin 2008, oral communication) who demonstrated that the anti-IL-13 mAb IMA-638 significantly inhibited allergen-induced early and late phase bronchoconstriction in patients with mild allergic asthma.

The aim of this study was to determine whether IL-13 has a causative role in the pathogenesis of airway inflammation, remodelling and dysfunction stimulated by chronic exposure of mice to inhaled HDM in the absence of adjuvant or peripheral sensitisation. Furthermore, we sought to determine the importance of IL-13 during both the development and maintenance of established airway pathology caused by chronic exposure to HDM. The aim of the study was met. Using an anti-IL-13 neutralising mAb we demonstrated that IL-13 is important for both the development and maintenance of airway pathology caused by pulmonary sensitisation to HDM.

## Results

### Neutralisation of IL-13 inhibits airway cellular inflammation

**Figure 1:**
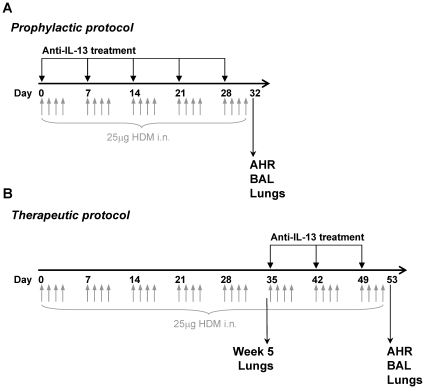
Experimental protocols for chronic exposure to house dust mite (HDM). (A) Mice received intranasal HDM on 4 consecutive days per week for 5 consecutive weeks with concomitant administration of anti-IL-13 mAb, isotype control or vehicle s.c. weekly (prophylactic treatment). (B) Mice received intranasal HDM on 4 consecutive days per week for 8 consecutive weeks with concomitant administration of anti-IL-13 mAb, isotype control or vehicle s.c. weekly starting on week 6 (therapeutic treatment). AHR and baseline lung function was assessed as R_L_ and C_dyn_ in terminally anaesthetised mice 24 hours after the last HDM instillation on day 32 (A) or day 53 (B). BAL and lungs were removed on day 32 (A) or day 53 (B) for assessment of airway inflammation and histological analysis. In addition, lungs were taken from an additional group on day 35 for histological analysis prior to therapeutic treatment (week 5 lungs, B).

**Figure 2:**
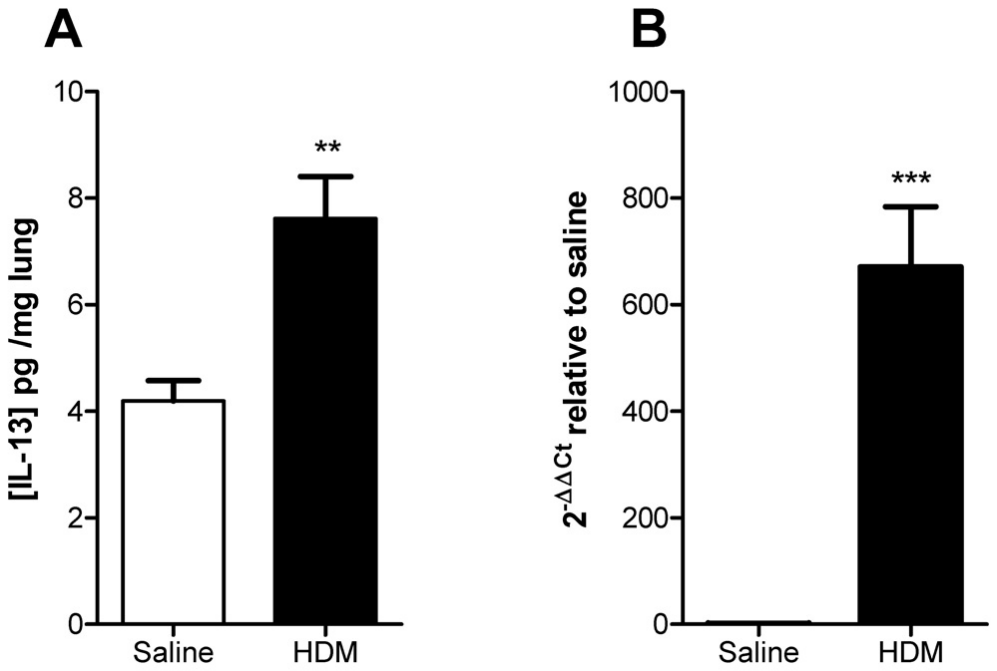
Up regulation of IL-13 in HDM sensitised mice. (A) IL-13 concentration in lung homogenate after 5 weeks of HDM sensitisation. (B) *IL-13* mRNA expression in lung tissue relative to saline sensitised mice. Data are shown as mean ± SEM, n = 8 mice per group. ** *p*<0.01, *** *p*<0.001 relative to saline group by unpaired t test.

**Figure 3:**
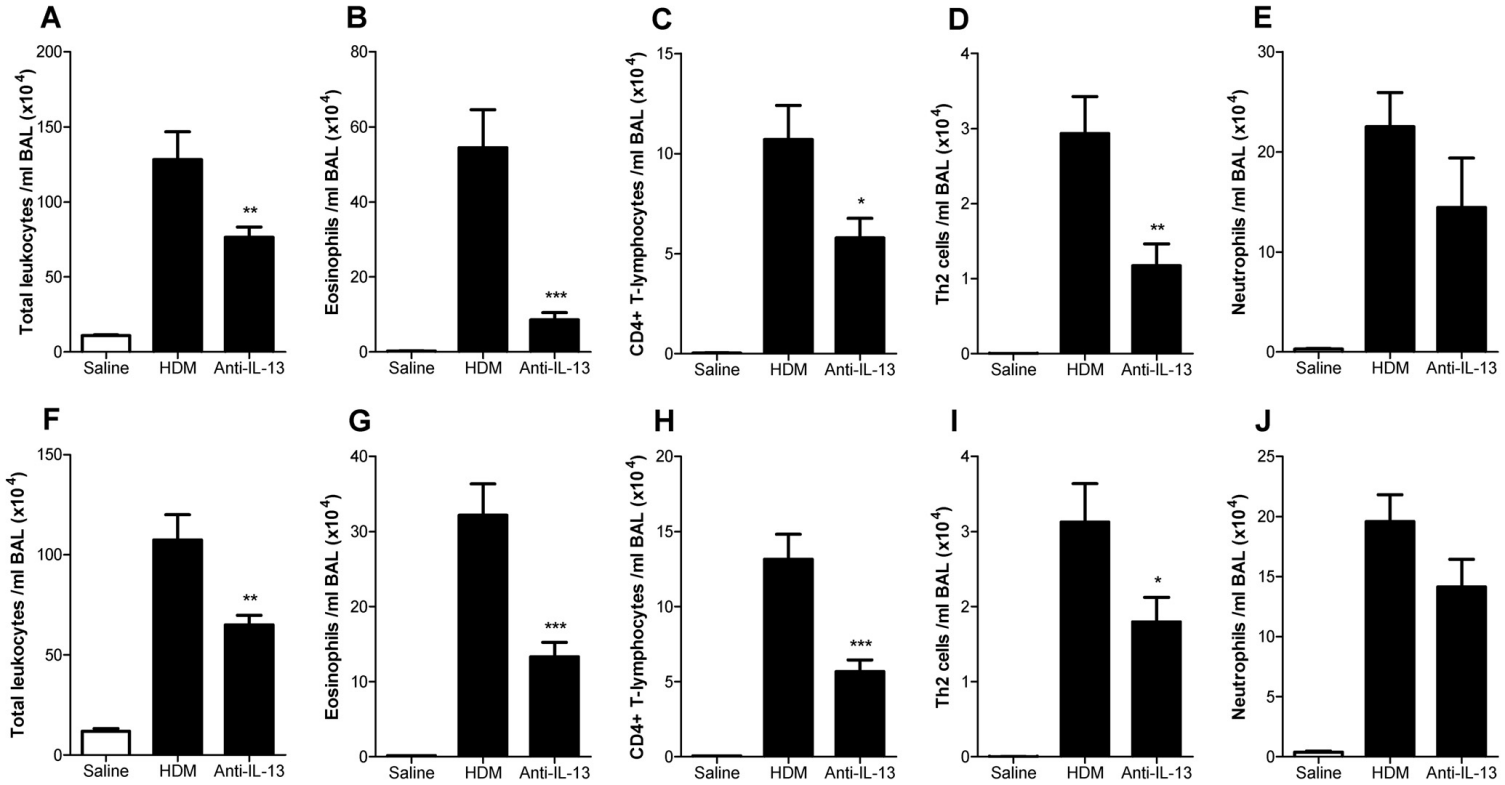
Effect of IL-13 neutralisation on BAL leukocytes. (A–E) Mice were treated prophylactically with anti-IL-13 mAb (10 mg/kg, s.c., weekly) administered immediately prior to and throughout HDM exposure. (F–J) Effect of IL-13 neutralisation on established airway inflammation. Mice were exposed to HDM for 8 consecutive weeks and treated with anti-IL-13 mAb therapeutically (10 mg/kg, s.c., weekly from week 6). Data are shown as mean ± SEM, n = 8 mice per group. *p<0.05, ** *p*<0.01, *** *p*<0.001 relative to HDM group by one-way ANOVA followed by Dunnett's post test.

IL-13 protein was upregulated in the lungs after 5 weeks of exposure to HDM, following the protocol in Figure 1A (4.19±0.38 pg/mg in mice exposed to saline; 7.62±0.79 pg/mg in mice exposed to HDM, *p*<0.01, Figure 2A). Similarly IL-13 mRNA was significantly elevated in mice exposed to HDM when compared to mice exposed to saline (670±113 fold, *p*<0.001, Figure 2B). Five consecutive weeks of exposure to HDM via the intra-nasal route stimulated cellular inflammation of the airways, observed as an elevation in BAL eosinophils, neutrophils and T-lymphocytes (Figure 3). To determine the role of IL-13 in the cellular inflammatory response, mice were treated prophylactically with anti-IL-13 mAb s.c. immediately prior to the first exposure to intranasal HDM and weekly s.c. thereafter. Prophylactic treatment with anti-IL-13 mAb (10 mg/kg) significantly inhibited total CD45^+^ leukocyte numbers in BAL fluid by 44% (Figure 3A) and significantly inhibited the number of siglec-F^+^ eosinophils in BAL by 85% (Figure 3B). Anti-IL-13 treatment significantly reduced total CD4^+^ T-lymphocyte numbers in BAL by 46% (Figure 3C) and T1ST2^+^ Th2 lymphocytes by 60% (Figure 3D). Neutrophil numbers in BAL were unaffected by treatment with anti-IL-13 mAb (Figure 3E).

To determine the role of IL-13 in maintaining the cellular inflammatory response in established disease, mice were exposed to HDM for 5 consecutive weeks prior to therapeutic administration of anti-IL-13 mAb (10 mg/kg, s.c., weekly), following the protocol in Figure 1B. Mice were subsequently exposed to HDM for a further 3 weeks, after which the BAL cellular infiltrate was characterised. Treatment with anti-IL-13 mAb using this therapeutic dosing regimen significantly reduced BAL CD45^+^ leukocytes by 44% (Figure 3F), siglec-F^+^ eosinophils by 59%; (Figure 3G), CD4^+^ T-lymphocytes by 57% (Figure 3H) and T1ST2^+^ Th2 lymphocytes by 43% (Figure 3I). No effects were observed on BAL neutrophil numbers (Figure 3J).

### Neutralisation of IL-13 inhibits mucus cell upregulation

**Figure 4:**
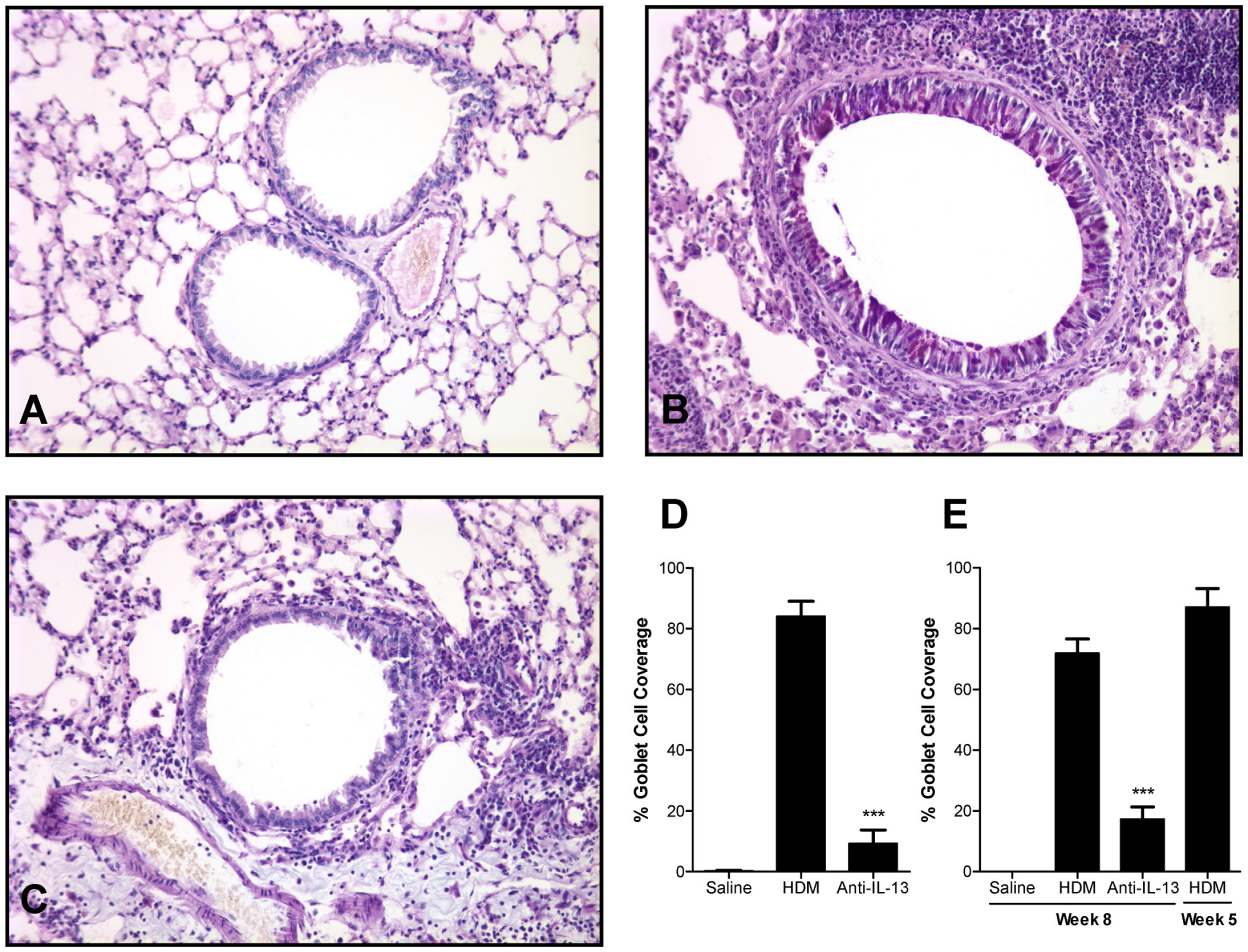
Effect of IL-13 neutralisation on epithelial goblet cell upregulation. (A–C) Representative photomicrographs of airway sections stained with AB/PAS (original magnification ×200) from mice exposed for 5 weeks to saline (A) or HDM (B–C) and treated prophylactically with vehicle (B) or anti-IL-13 mAb (C) (10 mg/kg s.c., weekly) immediately prior to and throughout HDM exposure. (D) Mice were treated prophylactically with anti-IL-13 mAb (10 mg/kg, s.c., weekly) administered immediately prior to and throughout the 5 consecutive weeks of exposure to HDM. (E) Effect of anti-IL-13 neutralisation on established goblet cell upregulation. Mice were exposed to HDM for 8 consecutive weeks and treated therapeutically with anti-IL-13 mAb (10 mg/kg, s.c., weekly from week 6) Week 5 HDM mice represent lung pathology immediately prior to therapeutic treatment, after 5 weeks of HDM exposure. Data are shown as mean ± SEM. n = 9–10 mice per group. *** *p*<0.001 relative to HDM group by one-way ANOVA followed by Dunnett's post test.

Airway mucus hypersecretion associated with goblet cell hyperplasia and metaplasia is a common feature of asthma that can be assessed in murine airway disease models by histological analysis of the airway epithelium. Intranasal administration of HDM for 5 consecutive weeks provoked a robust increase in the frequency of goblet cells within the airway epithelia, identified by AB/PAS staining (Figure 4B). Prophylactic treatment with anti-IL-13 mAb during the period of exposure to HDM significantly reduced goblet cell coverage (Figure 4D).

To investigate the role of IL-13 in maintaining elevated goblet cell number in established disease, mice were exposed to HDM for 5 consecutive weeks prior to commencement of treatment with anti-IL-13 mAb (10 mg/kg, s.c., weekly) and exposure to HDM for a further 3 weeks. As shown in Figure 4E, therapeutic treatment with anti-IL-13 mAb in established disease significantly reduced epithelial goblet cell coverage when compared to animals treated with vehicle.

### Neutralisation of IL-13 inhibits airway wall remodelling

**Figure 5:**
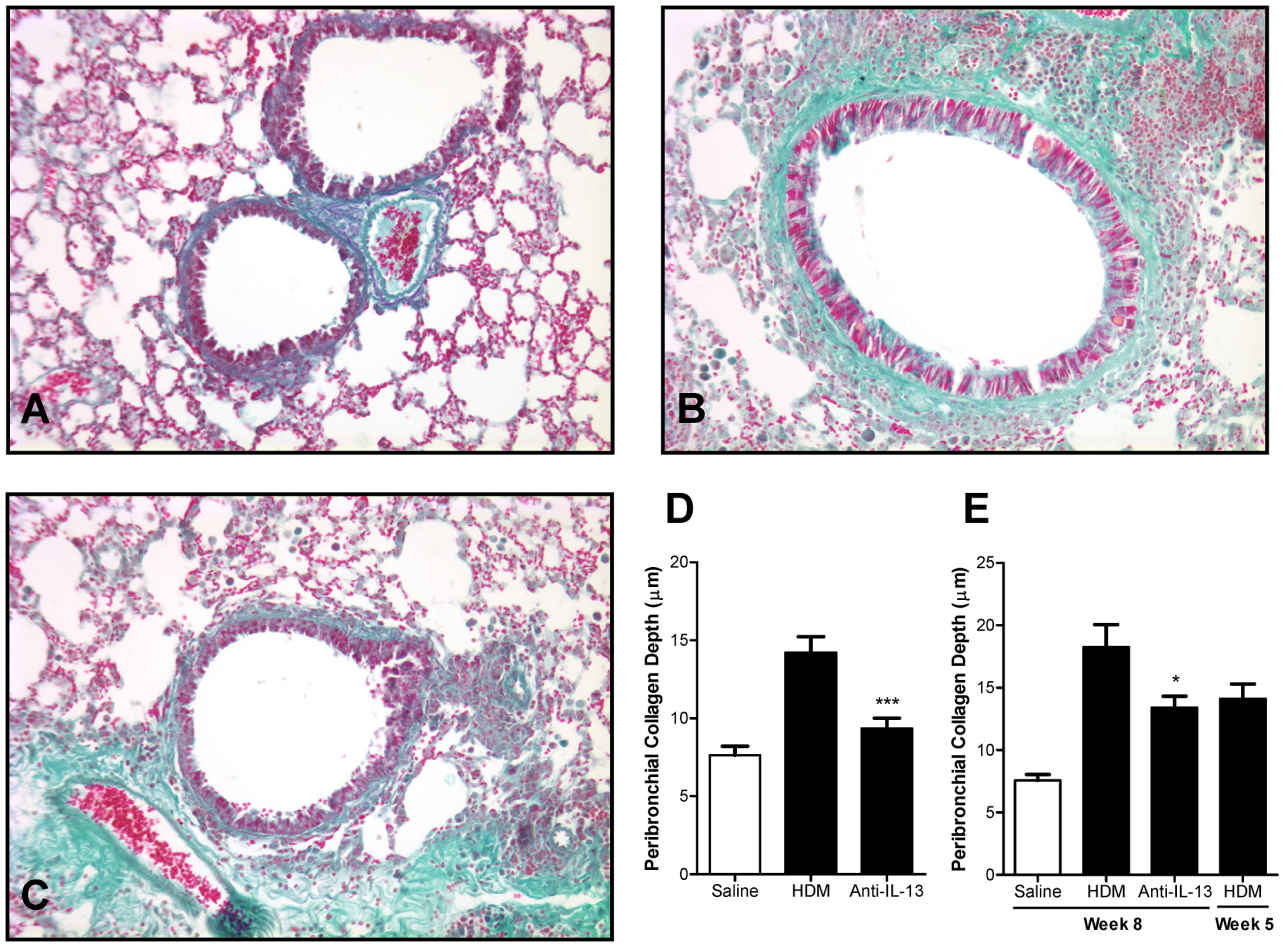
Effect of IL-13 neutralisation on peribronchial collagen thickness. (A–C) Representative photomicrographs of airway sections stained with Masson's trichrome (original magnification ×200) from mice exposed for 5 weeks to saline (A) or HDM (B–C) and treated prophylactically with vehicle (B) or anti-IL-13 mAb (10 mg/kg s.c., weekly) (C) immediately prior to and throughout HDM exposure. (D) Mice were treated prophylactically with anti-IL-13 mAb (10 mg/kg, s.c., weekly) administered immediately prior to and throughout the 5 consecutive weeks of exposure to HDM. (E) Effect of anti-IL-13 neutralisation in mice with an established increase in collagen thickness. Mice were exposed to HDM for 8 consecutive weeks and treated therapeutically with anti-IL-13 mAb (10 mg/kg, s.c., weekly from week 6). Week 5 HDM mice represent lung pathology immediately prior to therapeutic treatment, after 5 weeks of HDM exposure. Data are shown as mean ± SEM. n = 9–10 mice per group. * *p*<0.05, *** *p*<0.001 relative to HDM group by one-way ANOVA followed by Dunnett's post test.

Administration of HDM to the airways of mice for 5 consecutive weeks significantly increased the thickness of sub-epithelial collagen as visualised with Masson's trichrome stain (Figure 5B). These data support the observations of Johnson *et. al.* made in a similar mouse model using HDM [5]. Prophylactic treatment with anti-IL-13 mAb significantly inhibited the increase in airway sub-epithelial collagen thickness stimulated by repeated exposure to HDM (Figure 5D).

Peribronchial sub-epithelial collagen thickness was further significantly increased when exposure to HDM was extended from 5 to 8 consecutive weeks (Figure 5E). Therapeutic treatment with anti-IL-13 mAb (10 mg/kg, s.c., weekly) starting from week 6 significantly prevented the additional increase in peribronchial collagen thickness stimulated by continued exposure to HDM.

### Neutralisation of IL-13 inhibits AHR to methacholine and improves baseline R_L_ and C_dyn_

**Figure 6:**
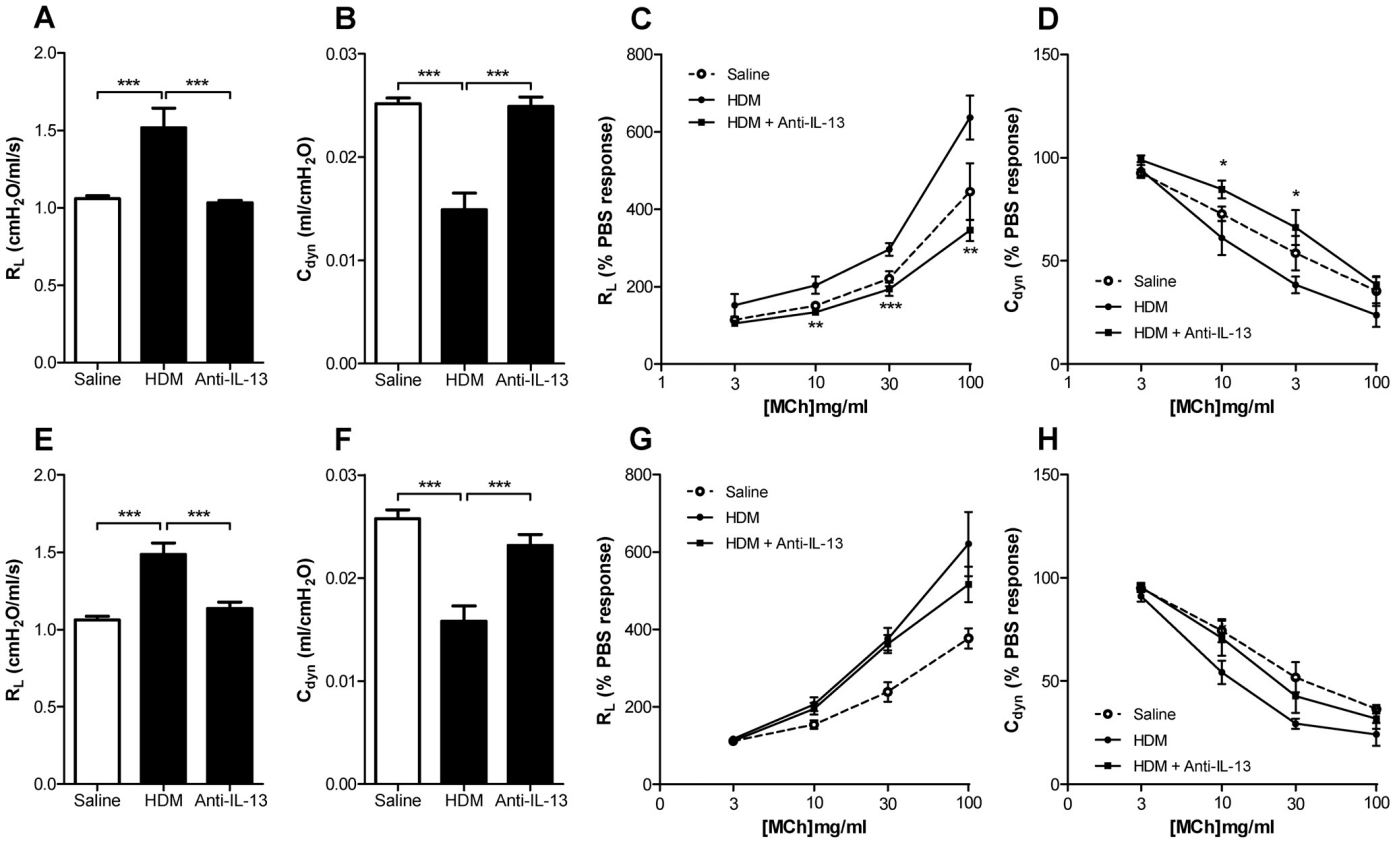
Effect of IL-13 neutralisation on lung function and AHR to inhaled MCh. AHR to MCh was determined as changes in lung resistance (R_L_) and dynamic compliance (C_dyn_) measured after 5 weeks (A–D) or 8 weeks (E–H) of exposure to HDM. (A–D) Mice were treated prophylactically with anti-IL-13 mAb (10 mg/kg, s.c., weekly) or isotype control administered immediately prior to and throughout the 5 consecutive weeks of exposure to HDM. (A, B) Baseline R_L_ and C_dyn_ were determined prior to MCh exposure. (C, D) Changes in R_L_ and C_dyn_ in response to MCh relative to PBS response. (E–H) Mice were exposed to HDM for 8 consecutive weeks and treated therapeutically with anti-IL-13 mAb or isotype control from week 6 (10 mg/kg, s.c., weekly). (E, F) Baseline R_L_ and C_dyn_ were determined prior to MCh exposure. (G, H) Changes in R_L_ and C_dyn_ in response to MCh relative to PBS response. Data are shown as mean ± SEM. n = 7–8 mice per group. * *p*<0.05, ** *p*<0.01, *** *p*<0.001 relative to HDM group by one-way ANOVA followed by Dunnett's post test.

We have determined the effect of IL-13 neutralisation on lung function in the HDM model by assessment of R_L_ and C_dyn_ at baseline and after methacholine exposure. Five consecutive weeks of intranasal HDM exposure caused a robust decline in lung function as shown by elevated baseline R_L_ and reduced baseline C_dyn_. Furthermore, HDM exposure resulted in hyperresponsiveness to inhaled methacholine demonstrated by percent change in R_L_ and C_dyn_ relative to PBS response (Figure 6). Prophylactic treatment with anti-IL-13 mAb (10 mg/kg, s.c. weekly) normalised baseline R_L_ and C_dyn_ to levels measured in animals exposed to saline (Figure 6A and 6B). Prophylactic treatment with anti-IL-13 mAb significantly and robustly inhibited AHR to inhaled methacholine (Figure 6C and 6D).

The therapeutic dosing protocol was employed to determine the role of IL-13 in maintaining changes in lung function in established disease (Figure 1B). Therapeutic treatment with anti-IL-13 mAb from week 6 of exposure to HDM significantly improved baseline R_L_ and C_dyn_ measured at week 8 (Figure 6E and 6F, respectively). However, therapeutic treatment with anti-IL-13 mAb from week 6 did not inhibit AHR to inhaled methacholine measured at week 8 (Figure 6G and 6H). In contrast to the robust effect of prophylactic anti-IL-13 mAb treatment on AHR to methacholine, therapeutic treatment did not inhibit AHR despite reversing changes in baseline lung function.

## Discussion

In this study we have demonstrated that neutralisation of IL-13 in mice prevents the development and persistence of pathological and functional changes in the airways caused by repeated exposure to HDM.

We have employed an experimental protocol using repeated, prolonged inhaled administration of HDM, in the absence of experimental adjuvants or peripheral sensitisation, to more faithfully model the exposure to allergen experienced by asthmatic patients. Previous studies have shown that chronic exposure of mice to HDM results in immunological changes and airway pathology similar to that observed in persistent asthma. Johnson *et.al.* report that chronic exposure of mice to inhaled HDM for 5 days per week for up to 7 consecutive weeks stimulated a robust helper T cell Type 2 (Th2) response, accumulation of inflammatory leukocytes in the lung parenchyma and airways, peribronchial collagen deposition, epithelial goblet cell upregulation and AHR to the non-specific inhaled spasmogen methacholine [5]. In the present study we demonstrate that repeated and continuous administration of HDM caused accumulation of proinflammatory effector leukocytes in the airway. Furthermore, in agreement with the observations of Johnson *et. al.* [5], we found that chronic exposure to HDM caused increased collagen deposition in the bronchial wall, epithelial goblet cell hyperplasia and AHR to methacholine. Chronic exposure of mice to HDM, without experimental adjuvant, resulted in significantly increased baseline lung R_L_, and decreased C_dyn_, measured 24 h after the last HDM challenge. This is a useful surrogate for the late asthmatic response and demonstrates how chronic exposure to HDM results in fundamental changes in airway dynamics.

Prophylactic and therapeutic treatment with a high affinity, potent, neutralising anti-IL-13 mAb significantly inhibited HDM-induced airway eosinophilia, goblet cell upregulation and peribronchial collagen deposition. In addition anti-IL-13 treatment significantly inhibited Th2 cell accumulation in the airway. These data are in agreement with previous reports identifying a central role for IL-13 in the generation and maintenance of the murine allergic airways response following adjuvant-enhanced systemic sensitisation and inhaled exposure to ovalbumin [22], [26], aspergillus [27] or house dust mite [28]. Our data presented herein are the first demonstrating the importance of IL-13 in maintaining airway pathology in a disease model established using a clinically relevant aeroallergen administered by the inhaled route only and without the use of experimental adjuvants or peripheral sensitisation.

In asthmatic patients AHR to inhaled spasmogens is an indicator of poor disease control, and is associated with other relevant outcome measures such as lung function and markers of lung inflammation [29]. It has been shown clinically in allergic asthmatic patients that neutralisation of IL-13 improves lung function following inhaled allergen challenge, but does not prevent AHR to methacholine (Gauvreau *et. al.*, European Respiratory Society Berlin 2008, oral communication). A similar lack of effect on AHR in asthmatic patients was observed using pitrakinra, an IL-4 receptor antagonist that blocks IL-13 and IL-4 binding to IL-4Rα [30]. In contrast, neutralisation of IL-13 has been shown to reduce AHR to inhaled spasmogens in mouse [22] and sheep [23] models of asthma. We observe that prophylactic treatment with anti-IL-13 mAb reduced AHR to methacholine, prevented the increase in baseline airway resistance, R_L_, and decrease in dynamic compliance, C_dyn_, measured 24 h after allergen challenge. The effect we observe on baseline R_L_ and C_dyn_ is consistent with that obtained in an ovalbumin-driven model in mice using IL-13R-Fc to neutralise IL-13 [26]. Therapeutic treatment with anti-IL-13 mAb however reversed the HDM induced changes in baseline R_L_ and C_dyn_ without effecting methacholine provoked AHR. In this study we observe differential effects of anti-IL-13 mAb on AHR to methacholine following prophylactic and therapeutic treatment. Our data support the existing evidence that anti-IL-13 mAb are effective in reducing AHR to inhaled spasmogens in preclinical models, but not AHR to inhaled spasmogens in asthma patients treated therapeutically. Furthermore our data support the evidence that IL-13 is important for the late phase decline in lung function in preclinical models and in asthma patients. There are therapeutics in clinical use, for example the anti-IgE mAb omalizumab, that do not affect AHR to methacholine in asthma patients, but do improve the late phase decline in lung function and reduce the number of disease exacerbations [31], [32]. We are currently conducting studies in a mouse model of asthma exacerbation to investigate the efficacy of anti-IL-13 mAb therapy on the decline in lung function and AHR caused by exposure to HDM with concomitant viral infection.

IL-13 may act directly on airway smooth muscle, causing airway hyperreactivity to inhaled spasmogens and increase baseline R_L_. Studies have shown that IL-13 can directly act on airway smooth muscle cells to increase contractility [33] and upregulate leukotriene receptors [34]. IL-13 has been shown to stimulate autocrine release of IL-5 from ASM, resulting in hyperresponsiveness sensitive to inhibition with an anti-IL-5 mAb [35]. In addition of effects on ASM contractility, the effect of IL-13 on baseline R_L_ and C_dyn_ may be attributable structural changes such as bronchial fibrosis, goblet cell hyperplasia, and associated mucus hypersecretion causing airway obstruction. Indeed, mucus plugs were observed in the airways of some mice following exposure to HDM (data not shown).

The chronic HDM model of asthma has enabled us to investigate the effect of anti-IL-13 mAb treatment on both mechanism and disease-relevant endpoints. The effect of anti-IL-13 treatment on mechanism-based endpoints, for example inhibition of airway eosinophilia, are important for understanding the pharmacology of anti-IL-13 mAb and are findings consistent with IL-13 stimulating local production of the eosinophil chemoattractant eotaxin [36]. We suggest that the robust efficacy of anti-IL-13 mAb on disease-relevant endpoints in this model indicates where the benefit of anti-IL-13 therapeutics may be seen in clinical practice. Thus, inhibition of chronic mucus hypersecretion, prevention of continued peribronchial collagen deposition and decreased airway wall compliance would represent effects of IL-13 neutralisation with the potential to fundamentally affect disease progression [37], [38].

In conclusion, these data demonstrate that IL-13 neutralisation reduces airway inflammation, normalises baseline lung function and reduces AHR to inhaled spasmogen in a mouse model of asthma driven by a clinically relevant aeroallergen administered by the inhaled route only, and without the use of experimental adjuvants. Our data support the proposition that neutralisation of IL-13 will benefit patients with chronic persistent asthma by reducing airway inflammation and improving lung function, with the potential to reduce the number and severity of exacerbations and the need for corticosteroids.

## Materials and Methods

### Antibody generation

Murinised anti-mouse IL-13 mAb (CA154_582, mAb A) was generated by UCB and *in vitro* activity was characterised as reported previously [39]. CA154_582 prevents the interaction between IL-13 and IL-13Rα1 and hence the subsequent association with IL-4Rα (K_D_ 11pM). In addition this anti-IL-13 mAb blocks IL-13 binding IL-13Rα2.

### Mice

Male Balb/c mice (20–25 g) were obtained from Harlan (UK). All mice were housed in specific pathogen free conditions, in standard cages and provided with food and water *ad libitum*. All experiments were performed in accordance with the UK Animals (Scientific Procedures) Act 1986.

### HDM sensitisation and treatment

Male Balb/c mice were exposed to 25 µg HDM protein (Greer, USA) in 25 µl sterile saline via the intra-nasal (i.n.) route under light Isoflurane anaesthesia for 4 consecutive days per week for 5 or 8 weeks. Control mice received 25 µl sterile saline administered i.n. under light Isoflurane anaesthesia for 4 consecutive days per week for 5 or 8 weeks. Mice were treated with 10 mg/kg anti-IL-13 mAb s.c. immediately prior to HDM administration on day 0 or 35 as indicated, and weekly thereafter. HDM sensitised control mice were dosed with control mouse IgG (10 mg/kg mAb 101.4) accordingly. Mice were killed 24 hours after the final HDM exposure. Where therapeutic dosing was employed an additional HDM sensitised group was killed on day 35 for lung histological analysis immediately prior to anti-IL-13 treatment (Figure 1).

### Measurement of lung IL-13 mRNA and protein

Lung tissue IL-13 mRNA levels were determined in medial lung lobes relative to 18S expression as previously described in detail [39]. IL-13 mRNA expression was normalised to that of lungs from animals exposed to saline. For determination of IL-13 protein in lung tissue, cranial lung lobes were homogenized at 50 mg tissue/ml in HBSS containing ‘complete’ protease inhibitor cocktail (Roche, UK), prior to centrifugation (20800× g, 20 min) and measurement of IL-13 in supernatant by ELISA (R&D Systems, UK).

### Flow cytometry

BAL sampling was performed post mortem via tracheal cannula with 3×0.4 ml PBS (containing 0.4% v/w BSA, 12 mM HEPES and 500 mM EDTA). BAL cell suspensions were stained with combinations of anti-mouse CD4, siglec F, Gr-1, CD45 (BD Biosciences, UK) and T1ST2 (MDbiosciences, USA). BAL samples were incubated with the antibody cocktail for 20 minutes on ice. BAL samples were fixed and red cells lysed with BD FACSlyse (BD Biosciences, UK) prior to centrifugation (400× g, 10 min, RT) and resuspension in PKH26 microbeads (Sigma, UK) for quantification of events acquired. Total leukocytes were identified as CD45^+^, CD4^+^ T-lymphocytes as CD45^+^ CD4^+^, Th2 cells as CD45^+^ CD4^+^ T1ST2^+^, eosinophils as CD45^+^ siglec F^+^ and neutrophils as CD45^+^ Gr-1^hi^. Flow cytometric acquisition was performed using the FACSCanto II system (BD Biosciences, UK) with offline analysis using Flowjo (version 8.8.4, Tree Star, USA).

### Histology

The left lung was excised post mortem and preserved in 10% formalin (Surgipath, UK), embedded in paraffin and sectioned for staining with Alcian blue periodic acid Schiff's stain (AB/PAS) for mucin or Masson's Trichrome for collagen. Goblet cell upregulation within the airway epithelia was assessed by measuring the length of airway basement membrane covered by goblet cells, expressed as a percentage of total basement membrane length in each airway. Three bronchioles were selected at random from each section; one section was analysed per mouse and the mean goblet cell coverage (%) was calculated for each mouse. Peribronchial collagen thickness was measured using Image-Pro Plus software (version 5, Media Cybernetics, USA). Three bronchioles were selected at random from each section and the mean depth of collagen from the basement membrane determined from five measurements around the bronchiole. One section was analysed per mouse and the mean collagen depth was calculated for each mouse.

### Measurement of Airway Hyperreactivity

AHR was determined by direct measurement of lung resistance (R_L_) and dynamic compliance (C_dyn_) in anaesthetised, ventilated mice using FinePointe apparatus (Buxco, USA). Twenty four hours after HDM instillation mice were terminally anaesthetised with ketamine (200 mg/kg, i.m.) and pentobarbital (35 mg/kg, i.p.), tracheotomised and ventilated at 160 breaths/min, tidal volume 200 µl. After a short period of acclimatisation baseline measurements of lung function were recorded. Mice were then exposed to aerosolised PBS and subsequently increasing concentrations of aerosolised methacholine via the tracheal cannula (3–100 mg/ml, Sigma, UK). AHR data is expressed as percent change from PBS response.

### Statistical analysis

Data are representative of two or more studies. Data are expressed as mean ± SEM, unless otherwise stated. Statistical significance was determined by parametric one-way analysis of variance (ANOVA) with Dunnett's post test. Differences from vehicle control are denoted by asterisks whereby * *P*<0.05, ** *P*<0.01 and ***P<0.001. Graph generation and statistical analyses were performed using GraphPad Prism (version 5.01).
